# The tumor promoter cysteinyl leukotriene receptor 1 regulates PD-L1 expression in colon cancer cells via the Wnt/β-catenin signaling axis

**DOI:** 10.1186/s12964-023-01157-6

**Published:** 2023-06-14

**Authors:** Shakti Ranjan Satapathy, Souvik Ghatak, Anita Sjölander

**Affiliations:** Cell and Experimental Pathology, Department of Translational Medicine, Clinical Research Center, Lund University, Skåne University Hospital, Jan Waldenströms Gata 35, 205 02 Malmö, Sweden

**Keywords:** Cysteinyl leukotriene receptor, PD-L1, Wnt/β-catenin, Colon cancer

## Abstract

Immunotherapy targeting programmed death-ligand 1 (PD-L1) or PD-1 in solid tumors has been shown to be clinically beneficial. However, in colorectal cancer (CRC), only a subset of patients benefit from PD-1/PD-L1 treatment. Previously, we showed that high cysteinyl leukotriene receptor 1 (CysLT_1_R) levels are associated with poor prognosis in CRC patients. Recently, we have revealed the role of the tumor promoter CysLT_1_R in drug resistance and stemness in colon cancer (CC) cells. Here, we show the role of the CysLT_1_R/Wnt/β-catenin signaling axis in the regulation of PD-L1 using both in vitro and in vivo preclinical model systems. Interestingly, we found that both endogenous and IFNγ-induced PD-L1 expression in CC cells is mediated through upregulation of CysLT_1_R, which enhances Wnt/β-catenin signaling. Therapeutic targeting of CysLT_1_R with its antagonist montelukast (Mo), as well as *CRISPR/Cas9*-mediated or doxycycline-inducible functional absence of CysLT_1_R, negatively regulated PD-L1 expression in CC cells. Interestingly, an anti-PD-L1 neutralizing antibody exhibited stronger effects together with the CysLT_1_R antagonist in cells (*Apc*^*mut*^ or *CTNNB1*^*mut*^) with either endogenous or IFNγ-induced PD-L1 expression. Additionally, mice treated with Mo showed depletion of PD-L1 mRNA and protein. Moreover, in CC cells with combined treatment of a Wnt inhibitor and an anti-PD-L1 antibody was effective only in β-catenin-dependent (*APC*^*mut*^) context. Finally, analysis of public dataset showed positive correlations between the PD-L1 and CysLT_1_R mRNA levels. These results elucidate a previously underappreciated CysLT_1_R/Wnt/β-catenin signaling pathway in the context of PD-L1 inhibition in CC, which might be considered for improving the efficacy of anti-PD-L1 therapy in CC patients.

Video Abstract

**Supplementary Information:**

The online version contains supplementary material available at 10.1186/s12964-023-01157-6.

## Introduction

Colorectal cancer (CRC) is the third most common cancer worldwide [1, 2], with increased lethality projected in the coming years. Both preclinical and clinical studies have indicated immune escape as one of the most important mechanisms by which cancer cells avoid elimination by encounters with the immune system and acquire resistance to antitumor drugs. One of the many mechanisms underlying tumor immune escape is mediated by programmed death receptor 1 (PD-1), a cell surface receptor encoded by the *PDCD1* gene that is expressed on different immune cells and acts as a receptor for programmed death ligands (PD-L1 and PD-L2) expressed on tumor cells [3]. The binding of PD-L1 to PD-1 triggers a negative feedback system that serves as a protective mechanism and is used by tumor cells to further increase PD-L1 expression and evade the host immune system. Research in the last two decades has improved our knowledge of the immune system’s capabilities, resulting in the development of immunotherapy [3]. As an antitumor strategy, immunotherapy has gained the undivided attention of cancer researchers worldwide due to its dramatic effects on clinical outcomes, especially in melanoma, non-small cell lung carcinoma, and breast carcinoma [4, 5]. The PD-1/PD-L1 signaling pathway has also been demonstrated to play a role in regulating the tumor microenvironment in colon cancer (CC) [6]. However, in CRC, promising outcomes have been achieved both in mouse and human studies only in certain contexts, such as mismatch repair (MMR) deficiency, more specifically termed the ‘MSI-high’ status, which is found in a small percentage of CRC patients [7, 8]. In contrast, the majority of CRC patients have a microsatellite-stable (MSS) or mismatch repair-proficient status and do not benefit from immune checkpoint blockade. Hence, targeting the commonly altered signaling pathways in CRC, such as the Wnt signaling pathway [9], might help to inhibit immunosuppression by targeting the regulation of immune checkpoint markers and ultimately facilitate better outcomes.

In the last few years, we [10–15] and others [16–20] have emphasized the importance of cysteinyl leukotriene (CysLT) receptors in the pathobiology of colorectal and other cancers. Cysteinyl leukotriene receptors are seven-transmembrane G protein-coupled receptors and are of two main types, CysLT_1_R and CysLT_2_R [21, 22]. CysLT_1_R’s high affinity ligand is leukotriene D_4_ (LTD_4_), and CysLT_2_R’s high affinity ligand is leukotriene C_4_ (LTC_4_). While CysLT_1_R acts as a tumor promoter, the expression of CysLT_2_R is associated with a better prognosis in colorectal cancer patients [12]. Our previous studies established the tumor-promoting role of CysLT_1_R in CC initiation and progression in both mice and humans [23–27]. Specifically, we found a tumor promoting role of CysLT_1_R in a mouse model of colitis, i.e., AOM/DSS-induced colitis-associated colon cancer (CAC), and in a model of spontaneous CC (*APC*^*Min/*+^). In addition, upon activation via LTD_4_, CysLT_1_R was shown to activate the ERK-MAPK signaling cascade and result in the translocation of active β-catenin into the nucleus in colon epithelial cells [28]. Moreover, in a recent report, we highlighted a positive feed-forward signaling loop between CysLT_1_R and Wnt/β-catenin that contributes to promoting 5-FU resistance and resistance-derived stemness in CC cells [29].

Here, we studied the role of CysLT_1_R signaling in regulating PD-L1 expression in preclinical models of CC. By using CC cell lines, we found that targeting CysLT_1_R reduce both Wnt/β-catenin, which inhibits both endogenous and IFNγ-induced PD-L1 expression. Notably, functional absence of CysLT_1_R in vitro and antagonism of CysLT_1_R in a mouse xenograft model were accompanied by downregulation of PD-L1. Moreover, treatment with a specific CysLT_1_R antagonist or a Wnt inhibitor was proven to be more effective in combination with anti-PD-L1 antibody treatment. Additionally, in the public datasets GSE39582 and TCGA-COAD, we found a significant positive correlation between *CYSLTR1* and *CD274* (PD-L1) expression at the transcriptional level. Our study reveals the importance of targeting CysLT_1_R to achieve beneficial outcomes of immune checkpoint blockade in CC patients.

## Methods

### Cell culture and treatment

All cell lines were purchased from the American Type Culture Collection (ATCC; NJ, USA) and were cultured in accordance with standard ATCC protocols. Unless otherwise stated, for all cell line-based experiments, HCT116 and HT-29 CC cells were cultured in McCoy’s 5A medium (HyClone™, GE Healthcare Life Sciences, USA), and SW480 and RKO CC cells were cultured in RPMI 1640 medium and MEM (Sigma Life Science, St. Louis, MO, USA) medium, respectively. All media were supplemented with 10% fetal bovine serum (FBS; HyClone™, GE Healthcare Life Sciences, USA), 1% L-glutamine, and 100 µg/mL penicillin–streptomycin solution. All cells were maintained in a humidified incubator with 5% CO_2_ at 37 °C. All cell lines were regularly monitored for mycoplasma contamination.

All experiments based on cultured CC cells were performed as follows: HT-29 and SW480 cells were pretreated with IFNγ (50 ng/mL) for 24 h. Next, the cells were treated with an anti-PD-L1 neutralizing antibody (atezolizumab, Atz; 20 ng/mL) alone or in combination with Mo pretreatment (Mo + Atz; 10 µM; 30 min) for 24 h. RKO cells were treated with 80 nM LTD_4_ for 24 h or pretreated with 10 µM Mo for 30 min and then treated with 80 nM LTD_4_ for 24 h. Cells were treated with the Wnt inhibitor XAV-939 (10 µM) for 6 h alone or together with atezolizumab (XAV-939 + Atz). After the required incubation period, cells were processed for extraction of either RNA or protein for the desired analysis.

### Antibodies and reagents

For western blotting, antibodies against the following proteins were used: PD-L1, CysLT_1_R, nonphosphorylated (active) β-catenin, total β-catenin, pSTAT1, STAT1, Lamin B1, and GAPDH. For immunofluorescence analysis, antibodies against PD-L1, nonphosphorylated (active) β-catenin, total β-catenin, and F-actin were used; for more details, see Table 1.

**Table 1:** List and details of reagents and antibodies used in this study

Reagents or Antibodies	Source	Identifier no	Dilution	Assay
PD-L1 (E1L3N®) XP® Rabbit mAb	Cell Signalling	13,684	1:1000	Immunoblotting
Recombinant Anti-PD-L1 antibody [28–8]	AbCam	ab205921	1:100	Immuno- fluorescence
Non-phospho (Active) β-Catenin (Ser33/37/Thr41) (D13A1) Rabbit mAb	Cell Signalling	8814	1:3000	Immunoblotting
Non-phospho (Active) β-Catenin (Ser33/37/Thr41) (D13A1) Rabbit mAb	Cell Signalling	8814	1:300	Immuno- fluorescence
Total β-Catenin (D10A8) XP® Rabbit mAb	BDBioscience	#8480	1:5000	Immunoblotting
CysLT1R	Novus Biologicals	NBP2-92,396	1:1000	Immunoblotting
Phospho-Stat1 (Tyr701) (58D6) Rabbit mAb	Cell Signalling	9167	1:1000	Immunoblotting
Stat1 (D1K9Y) Rabbit mAb	Cell Signalling	14,994	1:1000	Immunoblotting
Anti-GAPDH antibody (0411)	Santacruz	sc-47724	1:5000	Immunoblotting
Anti-Lamin B antibody (B-10)	Santacruz	sc-374015	1:5000	Immunoblotting
Alexa Fluor™ 546 Phalloidin	Invitrogen	A22283	1:400	Immuno- fluorescence
Goat anti-Rabbit IgG (H + L) Cross-Adsorbed Secondary Antibody, Alexa Fluor™ 647	Invitrogen	A32733	1:400	Immuno- fluorescence
DAPI	Sigma-Aldrich	D9542	1–5 μg/mL	Immuno- fluorescence
XAV-939	Selleck Chemicals	S1180	-	-
CHIR-99021	Tocris Bioscience	4423/10	-	-
Montelukast	Cayman Chemicals	35,779	-	-
Leukotriene D4	Cayman Chemicals	20,310	-	-
Atezolizumab (anti-PD-L1)	Selleck Chemicals	A2004	-	-
IFN-γ (human, recombinant)	PeproTech	300–02	-	-

### CRISPR/Cas9-based knockdown of *CYSLTR1*

CRISPR/Cas9-based knockdown of *CYSLTR1* was performed according to a previous protocol [29]. Briefly, 2.5 × 10^5^ cells/well were seeded in six-well tissue culture plates and incubated until 70–80% confluent. Cells were transfected with the *CRISPR/Cas9 CYSLTR1* plasmid (Santa Cruz Biotechnology, sc-416516) using Lipofectamine 3000 (Life Technologies, USA) for 48 h. After transfection, colonies were selected based on the GFP expression and were expanded. These cells were further used for the desired experiments in accordance with the manufacturer’s instructions. *CRISPR/Cas9-CTRL* (Santa Cruz Biotechnology, sc-410749)-transfected cells were used for comparison.

### Cell line with stable Dox-inducible *CYSLTR1* knockdown

The cell line with stable doxycycline (dox)-inducible *CYSLTR1* knockdown employed in the study was established with HCT116 cells by following a previously described protocol [29].

### Mouse xenograft model

HT-29, SW480, and HCT116 cell-based xenograft models were established in nude mice as described earlier [24, 25].

### Quantitative RT-PCR

Total RNA was extracted using a RNeasy Mini Kit (QIAGEN, 74,104) following the manufacturer’s protocol, and 2 μg of total RNA was used for complementary DNA (cDNA) synthesis using a RevertAid H Minus First Strand cDNA Synthesis Kit (Thermo Fisher Scientific, K1632). Quantitative RT-PCR was performed with the following TaqMan probes: *CD274* (PD-L1, Hs01125301_m1), *CYSLTR1* (CysLT_1_R, Hs00272624_s1), and *CTNNB1* (β-catenin, Hs00355045_m1). Gene expression was quantified relative to that of *HPRT1* (Hs99999909_m1), the housekeeping gene, using the ΔΔCt method, and all values were normalized to the corresponding value in the control group.

### Nuclear and cytoplasmic fractionation of CC cells

Fractionation of CC cells was performed using Nunc NE-PER™ nuclear and cytoplasmic extraction reagents (78,833‚ Thermo Fisher Scientific, UK) according to the manufacturer’s instructions. The protein concentrations in the nuclear and cytoplasmic fractions were estimated by the Bradford method, and the fractions were subjected to western blot analysis for the desired proteins of interest.

### Western blot analysis

Cell lysates were prepared using modified RIPA lysis buffer [30], and the protein concentration was estimated by the Bradford method. Approximately 30–40 µg of total protein was loaded into each well of 10% SDS–polyacrylamide gels, separated, and transferred onto PVDF membranes (Millipore, IPVH00010) using a wet transfer system operated at 100 V for 1 h. The membranes were incubated in blocking solution (5% nonfat milk or BSA in Tris-buffered saline containing 0.1% Tween 20) for 1 h before incubation with the primary antibodies of interest at 4 °C overnight. Horseradish peroxidase-conjugated, host species-matched secondary antibodies (DAKO, Denmark) were used. The dilutions of the primary and secondary antibodies are listed in Table 1. The blots were developed using an enhanced chemiluminescence (ECL) substrate (Millipore, WBULS0500) and visualized using a Bio-Rad ChemiDoc™ Imaging System (Bio-Rad, CA, USA). Bio-Rad Image Lab software was used for densitometric analysis.

### Immunofluorescence analysis

CC cells were fixed with 4% PFA for 10 min, permeabilized with 0.5% Triton X-100 for 15 min and blocked with 5% goat serum (DAKO, Denmark) or 5% BSA for 1 h. The cells were washed at least 3 times with 1X PBS (phosphate-buffered saline) after the fixation and permeabilization steps. Thereafter, the cells were incubated with the anti-PD-L1 antibody overnight at 4 °C and with a goat anti-rabbit secondary antibody and phalloidin for 1 h at room temperature. Nuclei were stained with DAPI (5 µg/mL) for 10 min at room temperature. After each staining step, the cells were washed three times with 0.1% PBS-T (1X PBS containing 0.1% Tween 20). Images were acquired using a confocal microscope (Zeiss LSM 700: Carl Zeiss Microscopy GmbH, Jena, Germany) with a 63X oil objective lens. All images were acquired using the same settings for laser power and detector gain for uniform comparisons. Images were analyzed and processed using LSM Zen software.

### CC patient material

Matched normal and tumor tissues were processed as described earlier [31]. Cell lysates were prepared using modified RIPA lysis buffer [30], and the protein concentration was estimated by the Bradford method.

### Public datasets and resources

This study used previously published public or restricted patient data. Genome-wide RNA sequencing data expressed as transcripts per million (TPM) values and clinical information associated with the corresponding COAD samples from The Cancer Genome Atlas (TCGA) database (https://portal.gdc.cancer.gov/; https://tcpaportal.org/tcpa/; ≤ June 20, 2020) [32] and gene expression microarray data from the GSE35982 dataset were downloaded. Data for a total of 327 and 585 patients in the TCGA-COAD and GSE35982 datasets, respectively, were included in the study. The gene expression data in the datasets were normalized using the TMM method and log2-transformed for further analysis.

### Statistics

All quantitative data are presented as the mean ± S.E.M. or mean ± S.D. of at least three independent experiments or biological replicates, as indicated in the figure legend for each experiment. Statistical analyses were performed using GraphPad Prism version 9.0 (GraphPad Software, Inc., San Diego, CA, USA) unless otherwise stated. P values were calculated as mentioned in the figure legends, and a two-tailed *P* value of < 0.05 was considered to indicate a statistically significant difference.

## Results

### Treatment with a CysLT_1_R-specific antagonist reduces endogenous and IFNγ-induced PD-L1 expression in CC cells

**Fig. 1:**
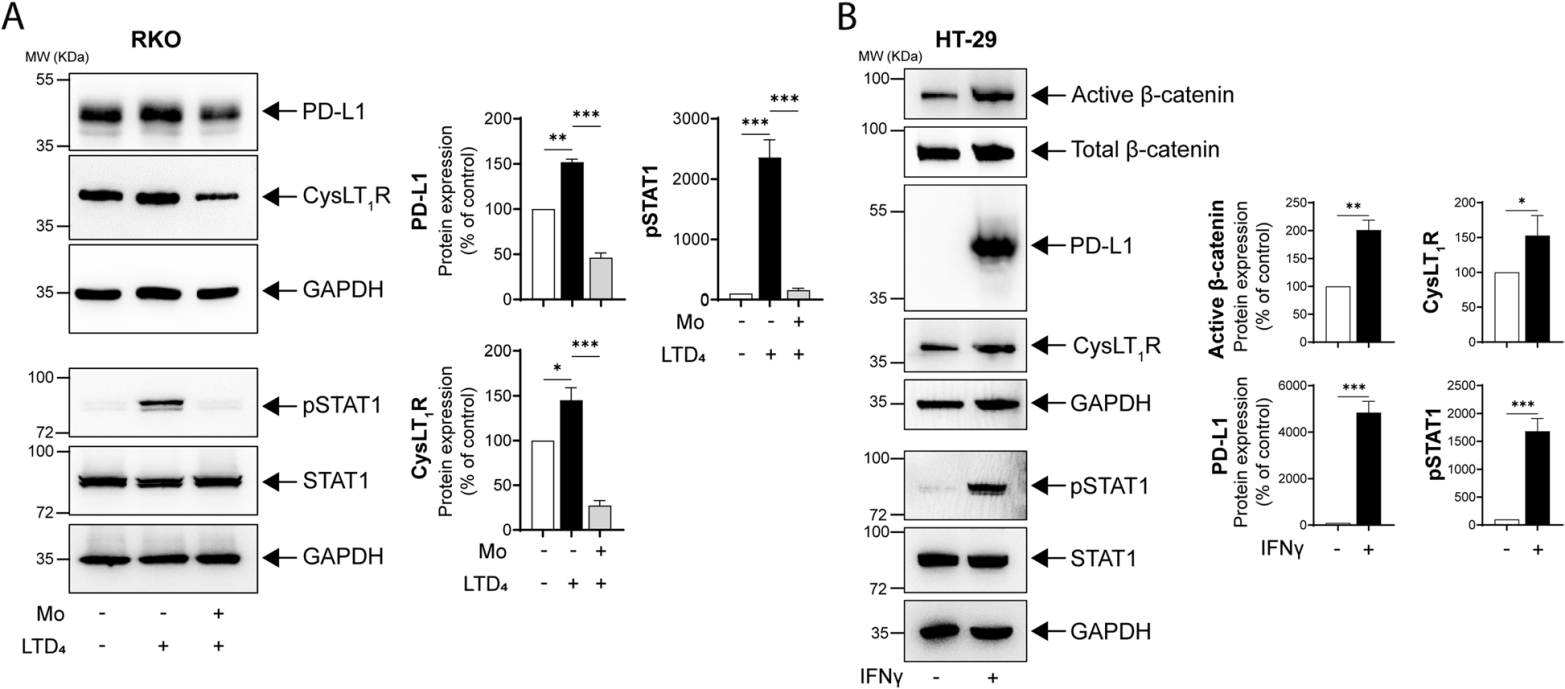
Treatment with the CysLT_1_R-specific antagonist Montelukast (Mo) reduces endogenous as well as IFNγ-induced PD-L1 expression in CC cells. **A** Western blots showing the expression of the indicated proteins in RKO CC cells stimulated with LTD_4_ (80 nM) with or without pretreatment with Mo (10 µM, 30 min). The blots are representative of three replicates, and the results are shown in the densitometry graphs. **B** Western blots showing alterations in the expression of the indicated proteins in HT-29 cells after IFNγ (50 ng/mL, 24 h) stimulation. The blots are representative of three replicates, and the results are shown in the densitometry graphs. In all the western blot panels, GAPDH served as the loading control. Mean ± SEM. *P < 0.05, ***P < 0.001, two-tailed unpaired t test. MW, relative molecular weight expressed in kilodaltons (kDa)

Most CC cell lines exhibit low PD-L1 expression, which requires IFNγ stimulation; considering tumor heterogeneity, it is also important to consider cells with endogenous PD-L1 expression. We first employed RKO CC cells, which exhibit endogenous PD-L1 expression, and stimulated CysLT_1_R with LTD_4_. We found a significant increase in PD-L1 expression, which was significantly reduced by pretreatment with the CysLT_1_R-specific antagonist montelukast (Mo) (Fig. 1A). Furthermore, we observed STAT1 phosphorylation in RKO cells after LTD_4_ stimulation, and this phosphorylation was also significantly reduced by Mo pretreatment (Fig. 1A).

Next, we checked PD-L1 expression in our other CC cell lines, all of which showed undetectable PD-L1 mRNA (*CD274*) and protein expression. We used MDA MB 231 triple-negative breast cancer cells, which exhibit high PD-L1 expression (data not shown), as a positive control. Considering the low expression of PD-L1 in most CC cell lines, we decided to induce PD-L1 expression with IFNγ treatment, as IFNγ upregulates PD-L1 expression in tumor cells [33]. We therefore stimulated CC cells with IFNγ, and IFNγ-stimulated HT-29 CC cells showed a ninefold increase in *CD274* mRNA expression, with concomitant increased expression of *CYSLTR1* and its downstream signaling molecule *CTNNB1* (β-catenin) (Supplementary Fig. S1). This was also in line with the positive correlation between *IFNG* and *CYSLTR1* expression found in both the TCGA-COAD and GSE39582 public datasets (Supplementary Fig. S2A, B). In the western blot analysis, we observed a > 40-fold increase in PD-L1 expression, a 1.5-fold increase in CysLT_1_R expression, and a twofold increase in active β-catenin protein expression in IFNγ-stimulated cells compared to nonstimulated control cells (Fig. 1B). The IFNγ-mediated PD-L1 stimulation of HT-29 cells was found to occur via STAT1 phosphorylation (Fig. 1B). In SW480 CC cells, we observed an increase in PD-L1 protein expression after IFNγ treatment, which occurred mostly in the membrane and cytoplasmic compartments (Supplementary Fig. S2C).

### Functional absence of CysLT_1_R negatively regulates PD-L1 expression in CC cells

**Fig. 2:**
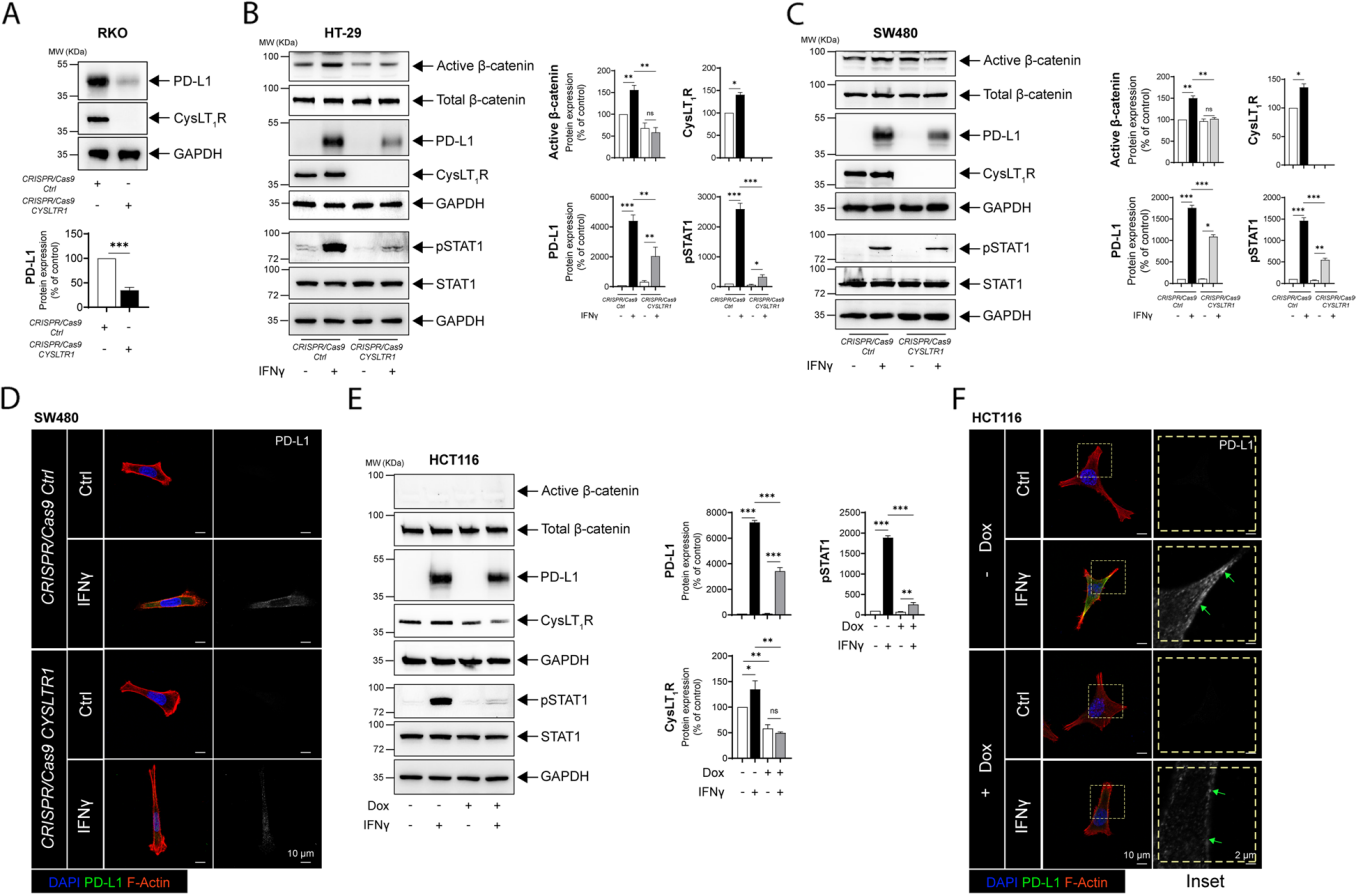
CysLT_1_R regulates PD-L1 expression in CC cells. **A** Western blots showing the expression of the indicated proteins in RKO cells transfected with *CRISPR/Cas9-Ctrl* or *CRISPR/Cas9-CYSLTR1*. The blots are representative of three replicates, and the results are shown in the densitometry graph. Western blots showing the expression of the indicated proteins in **B** HT-29, and **C** SW480 cells transfected with *CRISPR*/*Cas9-Ctrl* or *CRISPR/Cas9-CYSLTR1* with or without IFNγ stimulation. Densitometric comparison was made between unstimulated and IFNγ-stimulated cells. The blots are representative of three replicates, and the results for HT-29 and SW480 cells are shown in the densitometry graphs. Immunofluorescence images showing PD-L1 expression following treatment of **D** SW480 cells transfected with *CRISPR/Cas9-Ctrl* or *CRISPR/Cas9-CYSLTR1* prior to IFNγ stimulation. Bars, 10 μm. The right panel contains images showing the grayscale representation of PD-L1 expression in the different groups. **E** Western blot showing the relative expression of the indicated proteins in HCT116 cells with doxycycline (Dox)-inducible conditional knockdown of *CYSLTR1*. The blots are representative of three replicates, and the results are shown in the densitometry graphs. **F** Immunofluorescence images showing the expression of PD-L1 in HCT116 cells with doxycycline-inducible conditional knockdown of *CYSLTR1* with or without IFNγ stimulation. The magnified images are grayscale images (inset) of PD-L1 in the region of interest marked with the yellow dotted line. Bars, 10 μm or 2 µm as indicated in the images. In all the western blot panels, GAPDH served as the loading control. MW, relative molecular weight expressed in kilodaltons (kDa). Mean ± SEM. *P < 0.05, ***P < 0.001, two-tailed unpaired t test

The abovementioned results prompted us to further investigate the possible involvement of CysLT_1_R signaling in regulating PD-L1 expression in CC cells. To understand the importance of CysLT_1_R in CC cells with endogenous expression of PD-L1, we employed a CRISPR/Cas9 approach to specifically target the *CYSLTR1* gene and compared the modified cells with *CRISPR/Cas9-Ctrl*-transfected cells. LTD_4_ stimulation of CysLT_1_R elevates PD-L1 expression, and knockdown of *CYSLTR1* might negatively regulate PD-L1 protein expression. Indeed, functional deletion of *CYSLTR1* inhibited both endogenous and IFNγ-mediated PD-L1 upregulation in β-catenin-independent (RKO) and β-catenin-dependent (HT-29 and SW480) CC cells. While in RKO cells with functional deletion of *CYSLTR1*, PD-L1 expression was reduced twofold compared to that in *CRISPR/Cas9-Ctrl* cells, in HT-29 cells, PD-L1 expression was reduced 20-fold compared to the 40-fold increase in *CRISPR*/*Cas9-Ctrl* cells stimulated with IFNγ (Fig. 2A, B). Similarly, in SW480 cells, PD-L1 expression was reduced 1.5-fold in cells lacking *CYSLTR1* compared to the 20-fold increase in *CRISPR*/*Cas9-Ctrl*-transfected cells exposed to IFNγ (Fig. 2C). We previously showed that CysLT_1_R signaling induces β-catenin (Wnt/β-catenin) signaling activation [29]; here, we also showed depletion in the absence of *CYSLTR1*, suggesting a possible involvement of Wnt/β-catenin signaling. Additionally, in the immunofluorescence analysis, we found a decrease in the PD-L1 expression in *CRISPR/Cas9-CYSLTR1*-transfected SW480 CC cells stimulated with IFNγ compared to *CRISPR/Cas9-Ctrl*-transfected cells stimulated with IFNγ (Fig. 2D).

To further confirm the importance of CysLT_1_R in PD-L1 regulation in CC cells, we used a doxycycline-dependent *CYSLTR1* knockdown system in HCT116 cells. Interestingly, similar to the above observation, IFNγ-mediated induction of PD-L1 expression was limited (twofold) in β-catenin-independent HCT116 cells with *CYSLTR1* knockdown (Fig. 2E). Interestingly, consistent with the immunofluorescence results in SW480 cells, HCT116 cells with conditional knockdown of *CYSLTR1* showed a reduction in PD-L1 expression upon treatment with IFNγ compared with the corresponding control cells (Fig. 2F). This finding further strengthens the evidence supporting the role of CysLT_1_R in modulating PD-L1 expression.

Moreover, it was interesting to note that in HT-29 cells, the Mo-mediated decrease in IFNγ-induced PD-L1 expression was rescued in the presence of a GSK-3β inhibitor (CHIR-99021, 10 µM) (Supplementary Fig. 3A). When validating this observation in HT-29 cells with *CRISPR/Cas9*-based *CYSLTR1* knockdown, we noted that the depletion of PD-L1 expression due to the functional absence of *CYSLTR1* was also rescued by treatment with the GSK-3β inhibitor (CHIR-99021, 10 µM) (Supplementary Fig. 3B). This finding indicates the exclusive involvement of Wnt/β-catenin signaling in the regulation of PD-L1 expression in colon cancer cells.

### Antagonizing CysLT_1_R affects PD-L1 transcription and protein expression in mouse xenograft models

**Fig. 3:**
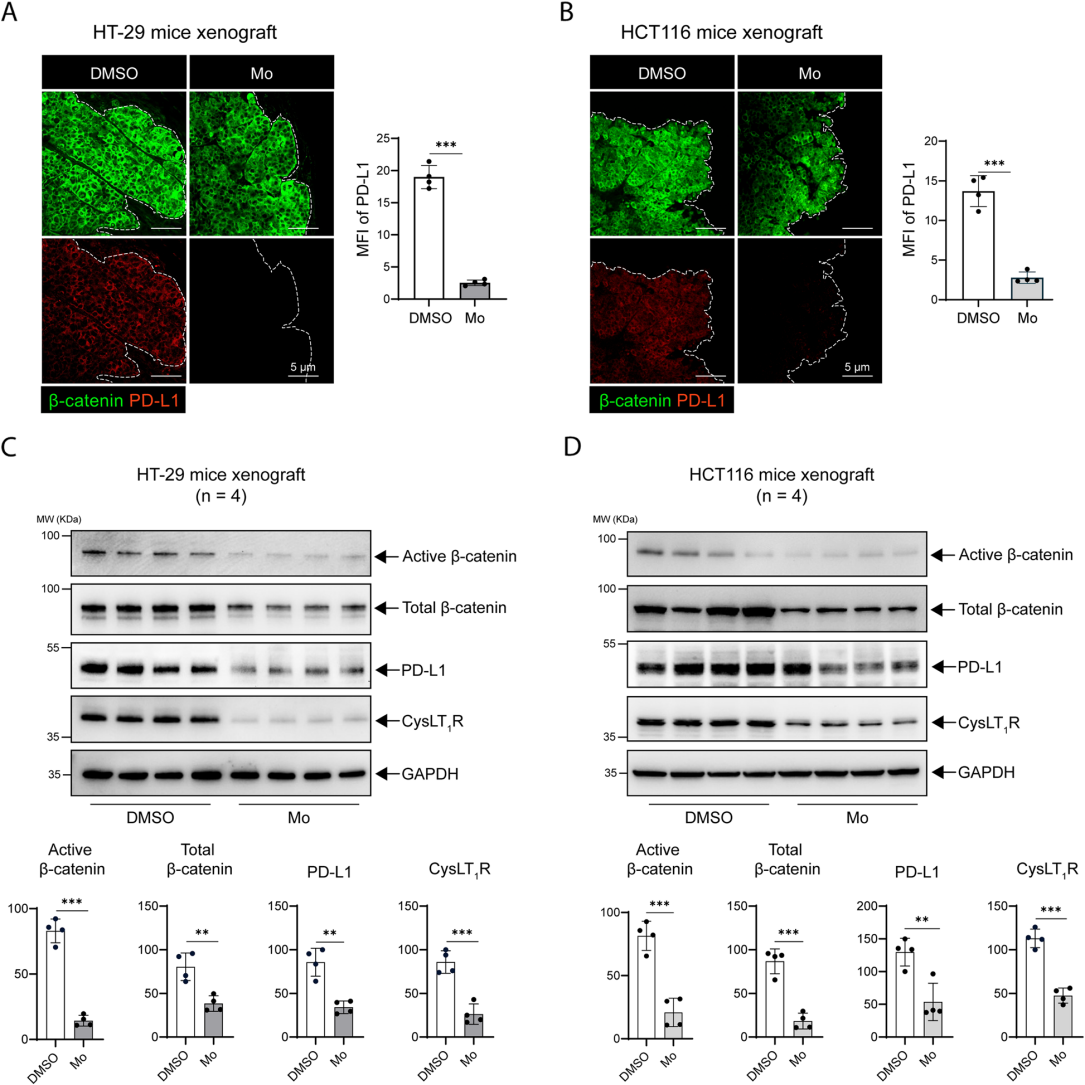
Antagonizing CysLT_1_R affects PD-L1 expression in different mouse xenograft models. Immunofluorescence images showing the expression of β-catenin (green) and PD-L1 (red) in **A** HT-29 and **B** HCT116 xenografts in mice treated with DMSO or Montelukast (Mo). Representative graphs showing the MFI (Mean fluorescence intensity) of PD-L1 (*n* = 4 mice per group in each xenograft condition). The scale bars are indicated in the images. The white dotted line marks the border of the xenograft section. Western blots of **C** HT-29 cell xenografts and **D** HCT116 cell xenografts. The blots show the expression of the indicated proteins of interest. Representative densitometric analysis results are shown in the graphs for HT-29 and HCT116 cells. In all the western blot panels, GAPDH served as the loading control. MW, relative molecular weight expressed in kilodaltons (kDa). The MFI in all confocal images was calculated with ImageJ software (NIH, USA). Mean ± SEM, *P < 0.05, ***P < 0.001, two-tailed unpaired t test

Next, to further evaluate the importance of CysLT_1_R as a potential therapeutic target, we used a human CC cell-derived xenograft mouse model and treated the mice with Mo, a CysLT_1_R-specific antagonist (Mo), or vehicle (DMSO). We found a significant decrease in the *CD274* transcript level in the Mo-treated groups compared to the DMSO-treated groups in both the HT-29 and SW480 cell-derived xenograft models (Supplementary fig. S4A). Notably, using immunofluorescence analysis, we observed that tissue sections from DMSO-treated mice contained significantly higher PD-L1 levels than those from Mo-treated mice in the HT-29 (Fig. 3A), HCT116 (Fig. 3B), and SW480 cell-derived xenograft models (Supplementary fig. S4B). The expression of β-catenin did not differ between the DMSO- and Mo-treated groups. However, in the protein extracts from the xenograft tissues, we observed a significant decrease in CysLT_1_R expression, with a concomitant decrease in the levels of active and total β-catenin in the groups treated with Mo compared to the DMSO-treated control groups in the β-catenin-dependent HT-29 and SW480 cell-derived and β-catenin-independent HCT116 cell-derived xenograft models (Fig. 3C, D; Supplementary Fig. S4C). In both the β-catenin-dependent (HT-29 and SW480) and β-catenin-independent (HCT116) xenograft models, PD-L1 expression was also significantly reduced after Mo treatment, indicating a positive regulatory impact of CysLT_1_R on PD-L1 expression in this CC model. Hence, similar to the effects in vitro, targeting CysLT_1_R also negatively affects PD-L1 expression in vivo.

### The CysLT_1_R-specific antagonist Mo shows stronger efficacy together with an anti-PD-L1 neutralizing antibody

**Fig. 4:**
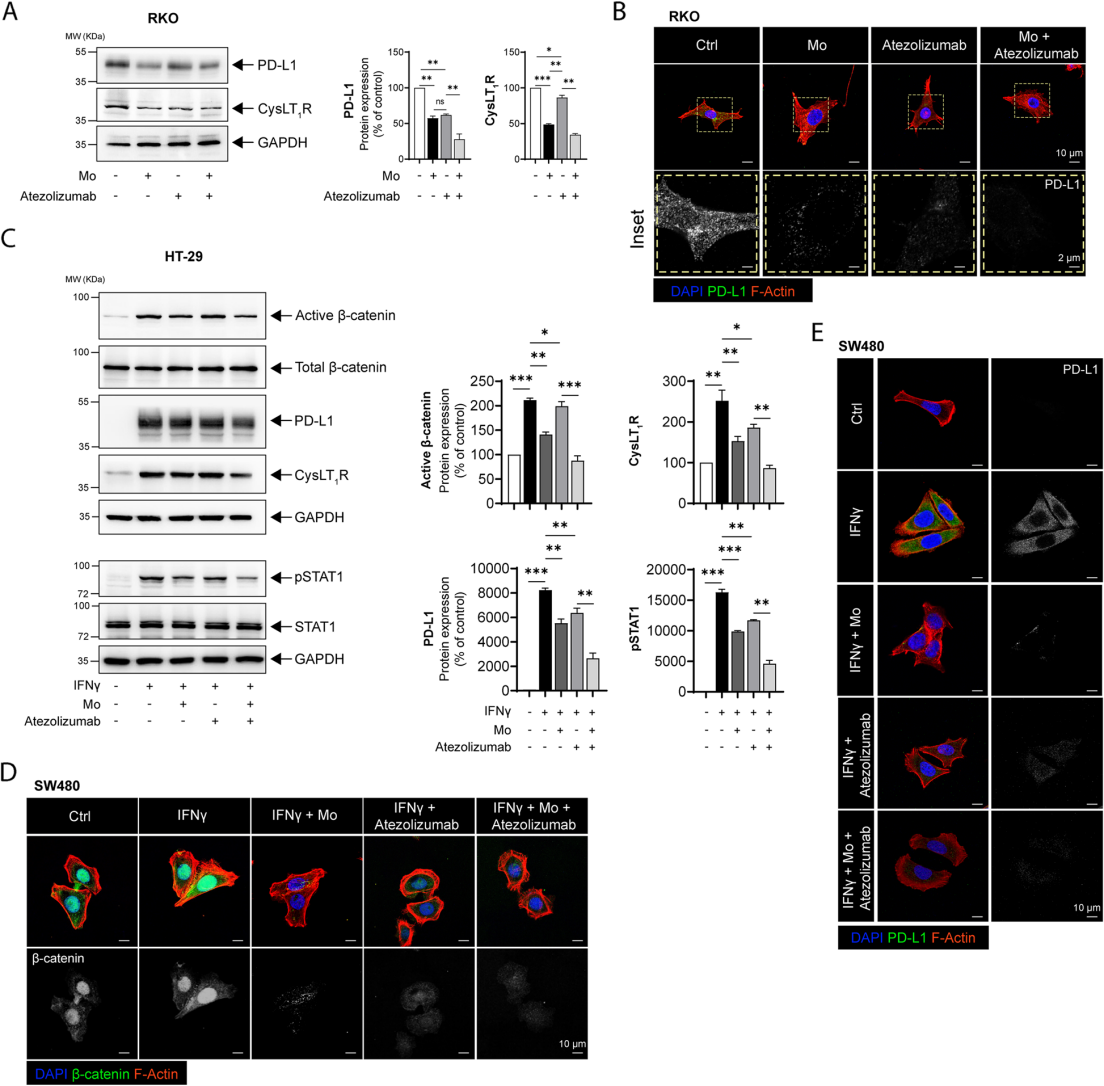
The CysLT_1_R-specific antagonist Mo shows combinatorial effects with an anti-PD-L1 neutralizing antibody. **A** Western blots showing the expression of the indicated proteins in RKO CC cells treated with Montelukast (Mo, 10 µM) or Atezolizumab (20 ng/mL) alone or together. The blots are representative of three replicates, and the results are shown in the densitometry graphs. **B** Immunofluorescence images showing PD-L1 expression in RKO cells. Cells were stimulated with Mo (10 µM) for 30 min or Atezolizumab (20 ng/mL) for 24 h alone or in combination with pretreatment with Mo (10 µM, 30 min) followed by Atezolizumab ( 20 ng/mL) for 24 h. The left panel shows the merged confocal images in each group with nuclear staining (DAPI, blue), PD-L1 staining (pseudocolored, green), and cytoskeleton staining (phalloidin, red), and the right column (insets) shows PD-L1 expression alone (grayscale) magnified from the region of interest marked by the yellow dotted line. The scale bars are indicated in the micrographs. **C** Western blots showing the expression of the indicated proteins in IFNγ-stimulated (50 ng/mL, 24 h) HT-29 cells subsequently treated with Mo or Atezolizumab alone or together (Mo + Atezolizumab). The blots are representative of three replicates, and the results are shown in the densitometry graphs. Immunofluorescence images showing the protein levels of **D** PD-L1 and **E** active β-catenin in IFNγ-stimulated SW480 cells subsequently treated with Atezolizumab alone or together with Montelukast (Mo + Atezolizumab). Bars, 10 µm. In all western blot panels, GAPDH served as the loading control. MW, relative molecular weight expressed in kilodaltons (kDa). Mean ± SEM. *P < 0.05, ***P < 0.001, two-tailed unpaired t test

Both anti-PD-1 and anti-PD-L1 antibodies have shown considerable promise in the treatment of solid tumors, including CRC [4, 5]. Although neutralizing antibodies have the best effect in cells harboring more mutations (MSI-high), the majority of CRC patients have a MSS status [7, 34]. Additionally, similar to patients with *Apc* mutations, CRC patients with high CysLT_1_R expression have a poor prognosis compared to the patients with low CysLT_1_R expression [12]. Therefore, we sought to determine whether a CysLT_1_R antagonist would exhibit improved efficacy in combination with a clinically approved anti-PD-L1 neutralizing antibody. We found that RKO cells treated with both Mo and Atezolizumab (anti-PD-L1 antibody) showed significantly lower PD-L1 expression than RKO cells treated with Mo or ﻿Atezolizumab alone (Fig. 4A). Furthermore, in cells treated with the Mo and ﻿Atezolizumab together, we observed reduction of CysLT_1_R, consistent with the decrease in PD-L1 expression (Fig. 4A). PD-L1 protein expression was also investigated by immunofluorescence imaging (Fig. 4B).

We next tested the combined treatment of CC cells with IFNγ-induced PD-L1 expression and observed that the mRNA level of *CD274* was significantly lower in cells pretreated with Montelukast (Mo) prior to Atezolizumab treatment, as were the transcript levels of *CYSLTR1* (CysLT_1_R) and *CTNNB1* (β-catenin) (Supplementary Fig. S5). In evaluating the effect of the combined treatment on the corresponding protein levels, we found a significant (twofold) decrease in PD-L1 expression in HT-29 cells treated with Mo and Atezolizumab  together (Fig. 4C), which was also consistent with the reduced STAT1 phosphorylation (Fig. 4C). The protein levels of CysLT_1_R and active β-catenin were decreased in cells treated with both Mo and ﻿Atezolizumab  compared with untreated cells but were higher than those in cells treated with Mo or ﻿Atezolizumab  alone. Consistent with the western blot results, we observed a decrease in the immunofluorescence of PD-L1 in SW480 cells (Fig. 4D). Moreover, immunofluorescence analysis of active β-catenin showed lower levels of active β-catenin in the cytoplasmic and membrane compartments in cells treated with both Mo and ﻿Atezolizumab, compared to its nuclear localization in both unstimulated control cells and IFNγ-treated control cells (Fig. 4E). This was further validated by western blot analysis of active β-catenin in the nuclear and cytoplasmic fractions of SW480 cells (Supplementary Fig. S6).

### Directly antagonizing the Wnt/β-catenin axis negatively impacts PD-L1 expression

**Fig. 5:**
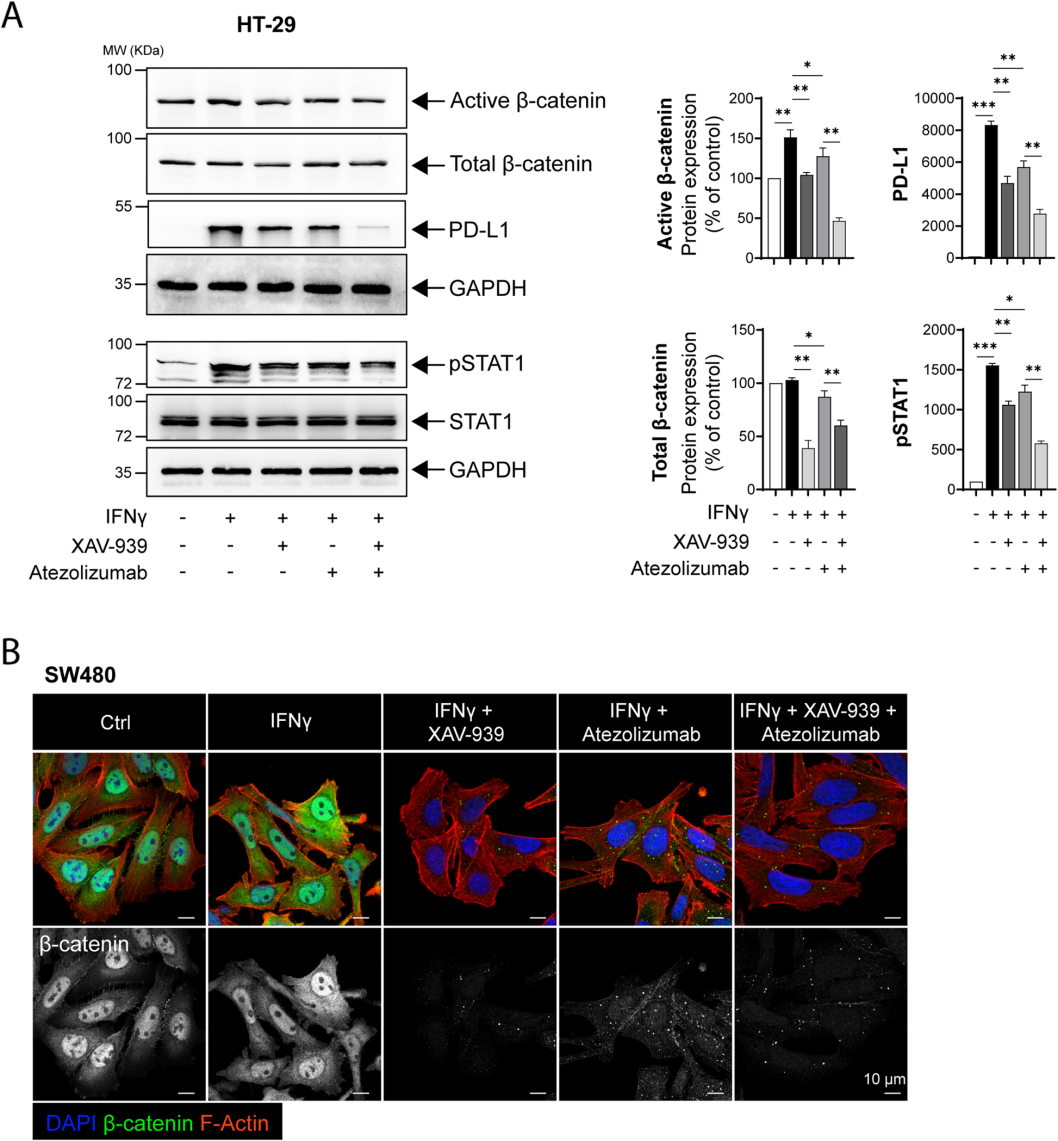
Directly antagonizing the Wnt/β-catenin axis negatively impacts PD-L1 expression. **A** Western blots showing the expression of the indicated proteins in HT-29 CC cells with or without prestimulation (IFNγ 50 ng/mL, 24 h). IFNγ-stimulated cells were further exposed to XAV-939 (Wnt inhibitor, 10 µM) or Atezolizumab alone or both together. The blots are representative of three replicates, and the results are shown in the densitometry graphs. **B** Immunofluorescence images showing the active β-catenin level in IFNγ-stimulated SW480 cells subsequently treated with XAV-939 (XAV) or Atezolizumab alone or in combination (XAV + Atezolizumab). Bars, 10 μm or 2 µm, as indicated in the images. The magnified images are grayscale images (inset) of active β-catenin in the region of interest marked with the white dotted line. In all the western blot panels, GAPDH served as the loading control. MW, relative molecular weight expressed in kilodaltons (kDa). Mean ± SEM. *P < 0.05, ***P < 0.001, two-tailed unpaired t test

Based on the above experiments in which we targeted the Wnt/β-catenin axis by antagonizing CysLT_1_R signaling, we next determined whether direct antagonism of the signaling results in a similar effect. Indeed, after targeting the Wnt/β-catenin signaling with the specific and effective tankyrase inhibitor XAV-939 [35] in β-catenin-dependent HT-29 cells (wild-type β-catenin), we found a significant decrease in IFNγ-induced PD-L1 expression, with a stronger effect achieved by the addition of the anti-PD-L1 antibody Atezolizumab compared to the individual effect of either agent (Fig. 5A). This was also in line with the significant reduction in the pSTAT1 protein level in XAV-939 + Atezolizumab-treated cells (Fig. 5A). Immunofluorescence analysis of active β-catenin showed a dramatic decrease in SW480 CC (wild-type β-catenin) cells treated with XAV-939 + Atezolizumab (Fig. 5B).

### PD-L1 expression positively correlates with CysLT_1_R and β-catenin expression in CC patients

**Fig. 6:**
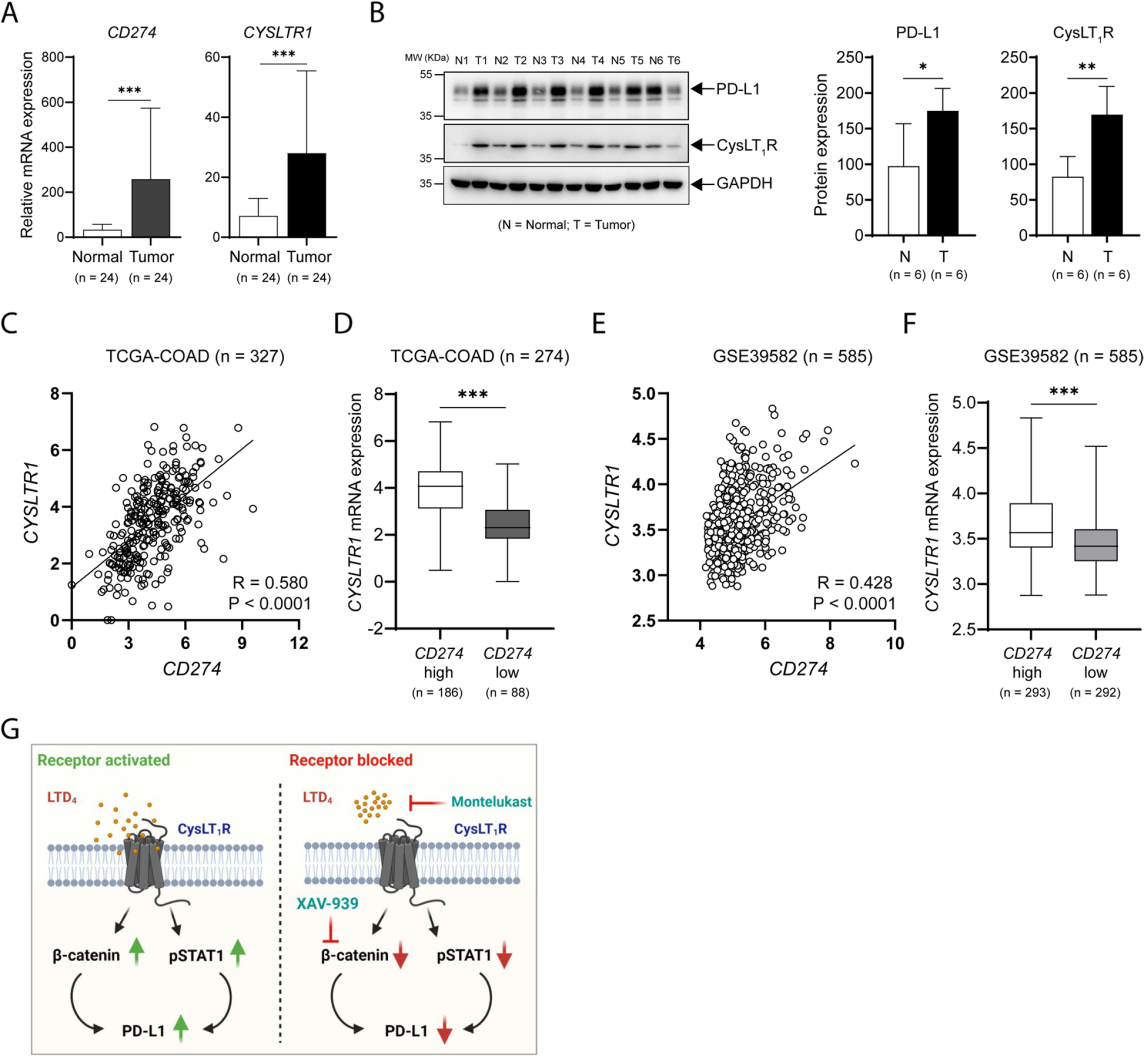
PD-L1 expression positively correlates with CysLT_1_R and β-catenin expression. **A** Bar graphs showing the relative mRNA expression levels of the indicated transcripts in matched normal and tumor tissues of CC patients (*n* = 24). **B** Western blots showing the expression of the indicated proteins in matched normal and tumor tissues of CC patients (*n* = 6). MW, relative molecular weight expressed in kilodaltons (kDa). Pearson correlation plots showing the positive correlation between the transcript levels of *CYSLTR1* and *CD274* in **C** TCGA-COAD cohort (*n* = 327) and **E** GSE39582 cohort (*n* = 585) of CC patients. Graphs showing *CYSLTR1* mRNA expression in CC patients with high or low *CD274* mRNA expression in the **D** TCGA-COAD cohort (*n* = 274) and **F** GSE39582 cohort of CC patients (*n* = 585). **G** Schematic summary showing alterations in the expression of the indicated proteins when CysLT_1_ is activated or blocked. The number of patients in each subgroup is shown in the figure. Mean ± SEM. *P < 0.05, ***P < 0.001, Mann‒Whitney U test

To compare the PD-L1 transcript (*CD274*) level between normal and tumor tissues in patients with CC, we employed the TCGA-COAD cohort (*n* = 327) and found higher expression of *CD274* in tumor tissues than in normal tissues (Supplementary Fig. S7). We validated our finding using 24 paired normal and tumor tissue samples and found significantly elevated *CD274* mRNA expression in the tumor tissues compared to the corresponding normal tissues (Fig. 6A); this pattern was also observed for *CYSLTR1* mRNA expression (Fig. 6A). Next, we analyzed lysates from 6 paired samples of normal and tumor tissue and observed significant increases in both PD-L1 and CysLT_1_R protein expression in the tumor tissues compared to the normal tissues (Fig. 6B). We also further validated our results in two different public datasets of CC (TCGA-COAD, *n* = 327, Fig. 6C, D; GSE9582, *n* = 585, Fig. 6E, F) and found a significant positive correlation between *CD274* (PD-L1) and *CYSLTR1* (CysLT_1_R) expression at the transcriptional level in both datasets. Additionally, we observed that patients with high *CD274* expression had high *CYSLTR1* expression and that patients with low *CD274* expression had low *CYSLTR1* expression (Fig. 6D, F).

Taken together, our findings indicate that the activation of CysLT_1_R in CC cells leads to upregulation of PD-L1 expression via STAT1 phosphorylation as well as activation of the Wnt/β-catenin signaling axis (Fig. 6G). Therapeutically antagonizing this receptor or the Wnt/β-catenin axis negatively regulates PD-L1 expression, which could be a beneficial clinical approach.

## Discussion

We previously reported elevated levels of CysLT_1_R in 5-FU-resistant CC cells, suggesting its involvement in drug resistance and resistance-derived stemness [29]. In addition, we determined that activation of Wnt/β-catenin signaling is involved in CysLT_1_R-mediated drug resistance of CC cells. Targeting Wnt/β-catenin signaling via CysLT_1_R signaling reduced 5-FU resistance and resistance-derived stemness. Moreover, we showed that activation of CysLT_1_R by IL-4 promotes stemness in colonospheres [24]. Here, we investigated the regulatory role of CysLT_1_R in CC cells and the impact of targeting CysLT_1_R signaling on PD-L1 expression.

Coordinated interactions between tumor cells and cells in the tumor microenvironment contribute to cancer progression. Tumor epithelial cells secrete cytokines, chemokines, and proinflammatory eicosanoids that recruit and reprogram various proinflammatory leukocytes to establish an immunosuppressive tumor microenvironment. Extrinsic IFNγ can upregulate PD-L1 expression in various cancer cells, including CC cells [33, 36]. In this study, we explored the effect of IFNγ on PD-L1 expression in vitro*,* and along with an increase in PD-L1 expression, we observed a concomitant increase in the CysLT_1_R expression level in CC cells that was inhibited in Mo-treated cells. The impact of IFNγ on CysLT receptors, specifically CysLT_1_R, has been shown by various researchers, including us, in immune cells [37], bronchial smooth muscle cells [38], and cancer models [18, 23]. In a uveal melanoma model, Slater et al. reported a significant increase in IFNγ levels in patients with high CysLT_1_R levels and a significant decrease in IFNγ levels in patients with low CysLT_1_R levels [18]. Additionally, in a mouse model of AOM/DSS-induced CC, we previously showed a decreased level of IFNγ in mice lacking *Cysltr1* [23]. IFNγ-mediated PD-L1 activation in various cancers occurs via STAT1 phosphorylation [33]. In addition, activation of CysLT_1_R can result in STAT1 phosphorylation, as evident in brachial epithelial cells, cells with abundant levels of CysLT_1_R and as shown here in CC cells [38]. Despite the positive correlation between IFNγ and CysLT_1_R expression as well as their sharing of common signaling pathways (via pSTAT1), it has not been shown that IFNγ-mediated activation of PD-L1 also occurs via activation of CysLT_1_R.

The Wnt/β-catenin signaling pathway is evolutionarily conserved and regulates many biological processes [39, 40]. Under physiological conditions, in the absence of Wnt ligands (inactive pathway), cytosolic β-catenin is targeted for degradation via phosphorylation by the destruction complex, and upon Wnt ligand binding, β-catenin translocates into the nucleus and promotes the transcription of the Wnt-responsive genes TCF/LEF [40]. Hyperactivation of Wnt pathways has been associated with many cancers, including CRC. In CRC, hyper-activation of Wnt/β-catenin signaling is mostly due to adenomatous polyposis coli (APC) mutations, which are found in approximately 90% of patients [41]. Hence, activation of this pathway is considered an indicator of poor survival in CRC patients because of therapy-induced resistance, stem cell enrichment or relapse. With respect to the immune checkpoints in cancer, numerous studies have shown an association of β-catenin with PD-1 or PD-L1 in different cancer models [42–45]. Recent reports showed β-catenin-mediated transcriptional regulation of PD-L1 [44] and indicated that targeting the Wnt/β-catenin pathway proved beneficial in glioblastoma [45]. In models of triple-negative breast cancer, it was shown that high PD-L1 expression was associated with stemness owing to activated Wnt signaling [43]. In addition, Wnt/β-catenin signaling have been reported to be an immunomodulator [42, 46]. The deep involvement of the Wnt/β-catenin pathway in multiple cellular processes makes it an attractive therapeutic target [47]. Here, we employed a Wnt pathway inhibitor (XAV-939) that specifically targets tankyrase, which generally hinders the ubiquitination of cytosolic β-catenin by binding to the destruction complex [35]. Interestingly, we found a stronger effect of atezolizumab and XAV-939 together, especially in cells harboring *APC* mutations (HT-29 and SW480) and dependent on β-catenin signaling (Fig. 5).

We previously showed that patients with high CysLT_1_R expression also had elevated nuclear β-catenin levels and lower levels of membrane β-catenin, which is associated with poor prognosis [12]. In two mouse models of CC (AOM/DSS and *APC*^*min*^), we reported a decrease in β-catenin expression in mice lacking CysLT_1_R expression compared to their wild-type littermates. Moreover, in colon epithelial cells and CC cell lines, we found LTD_4_-mediated nuclear translocation of active β-catenin, which was antagonized by Mo [28]. Furthermore, in a model of 5-FU resistant CC, we noted upregulation of β-catenin in the untreated cells, which was not seen in Mo-treated 5-FU resistant cells [29]. We have also earlier shown that LTD_4_ via CysLT_1_R signaling similar to Wnt signaling increases the level of active β-catenin which activates the TCF-LEF promotor activity via PI-3 kinase and GSK3β inhibition [48]. Similarly, in mouse embryonic stem cells, it has been reported that LTD_4_/CysLT_1_R signaling results in the STAT3 and GSK-3β phosphorylation but inhibition of β-catenin phosphorylation [49].

Here, we report that the IFNγ-mediated increase in PD-L1 expression also increased CysLT_1_R expression as well as the level of active β-catenin in CC cells. These results were also observed in RKO CC cells, which harbor *CTNNB1* mutations and exhibit endogenous expression of PD-L1. From this, it can be inferred that β-catenin could be the intermediate protein in CysLT_1_R-mediated PD-L1 regulation in CC cells. In contrast, Cen et al. [9] recently showed a link between the Wnt/β-catenin pathway and PD-L1 expression in CC cells. Using constitutive activation of β-catenin, they showed the β-catenin dependency of PD-L1 regulation in CC cells. Our study complements this report by revealing that CysLT_1_R is the upstream regulator of Wnt/β-catenin signaling-mediated PD-L1 regulation. Moreover, our findings identified the important role of CysLT_1_R in regulating PD-L1 expression in both β-catenin-dependent (*APC*^*mut*^ HT-29 and SW480) and β-catenin-independent (*APC*^*WT*^, *CTNNB1*^*mut*^ RKO and HCT116) CC cells.

Although patients with other solid tumors have benefited from immune checkpoint blockade therapy targeting the PD1/PD-L1 interaction, CRC patients did not benefit until the introduction of the concept of mismatch repair (MMR) proficiency or deficiency [7, 8]. MMR deficiency is found in only a small percentage of CRC patients [50], but patients with the MMR-deficient status (high level of somatic mutations) [51, 52] have been found to benefit from anti-PD-1/anti-PD-L1 treatment compared to their MMR-proficient counterparts due to the ability of the human immune system to recognize and neutralize the neoantigens produced as a result of MMR deficiency [5]. On the other hand, the majority of patients with CRC exhibit *APC* mutation. Similarly, CRC patients with high CysLT_1_R expression have a poorer prognosis. Hence, targeting CysLT_1_R/Wnt/β-catenin signaling could be beneficial for the majority of CRC patients. Therefore, instead of addressing the neoantigen issue in MSS patients, we sought to target the regulation of the protein expression of the desired immune checkpoint marker (PD-L1) in CC cells.

Based on this background information, we studied CC cells with either the MSS or MSI status. Both MSS (HT-29) and MSI (HCT116) cells with IFNγ-stimulated PD-L1 expression responded similarly to Mo treatment, with noticeable decreases in PD-L1, CysLT_1_R, and active β-catenin protein levels. Additionally, similar promising results were observed in RKO (MSI) CC cells with endogenous PD-L1 expression. This finding indicates that the efficacy of targeting the CysLT_1_R/Wnt/β-catenin signaling axis could provide benefits to patients with CRC with any MMR status. In addition, the combination of the anti-PD-L1 neutralizing antibody Atezolizumab with the CysLT_1_R antagonist Mo demonstrated a combinatorial effect in preclinical models of CC, emphasizing the power of combination therapy to promote the success of immunotherapy in CRC. Future research is required to study the impact of CysLT_1_R on the survival of patients with CRC based on PD-L1 expression, considering previous observations of its prognostic significance.

In summary, our study can be considered to advance the therapeutic approaches for CRC, highlight the important role of CysLT_1_R in regulating PD-L1 expression in CC cells, and elucidate the specific mechanism involved (Fig. 6G). Our results will further increase our understanding of the mechanism of action of this tumor promoter, which will strongly validate its candidacy as a potential therapeutic target for combinatorial effects when combined with the current immune checkpoint blockade regimens in patients harboring mutations in either *APC* or *CTNNB1*.

## Conclusion

Although CysLT_1_R is being targeted in various diseases [20], knowledge of its involvement in PD-L1 regulation might facilitate the development of targeted therapy that would benefit patients with high PD-L1 expression. This combinatorial effect could benefit CRC patients.

## Supplementary Information

**Additional file 1.**

## Data Availability

Data associated with the current study are available from the corresponding authors upon reasonable request.

## Abbreviations

CRC: Colorectal cancer
CC: Colon cancer
PD-L1: Programmed death ligand 1
PD-1: Programmed death receptor 1
CysLT_1_R: Cysteinyl leukotriene receptor 1
IFNγ: Interferon gamma
