# Supplementary material for: The tumor promoter cysteinyl leukotriene receptor 1 regulates PD-L1 expression in colon cancer cells via the Wnt/β-catenin signaling axis
Source: Cell Commun Signal. 2023 Jun 14;21:138. doi: 10.1186/s12964-023-01157-6 (PMC10265834; doi:10.1186/s12964-023-01157-6)

Supplementary file

The tumor promoter cysteinyl leukotriene receptor 1 regulates PD-L1 expression in colon cancer cells via the Wnt/β-catenin signalling axis

Shakti Ranjan Satapathy*, Souvik Ghatak, Anita Sjölander*

Cell and Experimental Pathology, Department of Translational Medicine, Lund University, Skåne University Hospital, Malmö, Sweden.

Running title: $CysLT_1R$ regulates PD-L1 expression in colon cancer.

*Address all correspondence to:

Shakti Ranjan Satapathy*, E-mail: shakti_ranjan.satapathy@med.lu.se

Anita Sjölander*, E-mail: anita.sjolander@med.lu.se

Cell and Experimental Pathology, Department of Translational Medicine,

Clinical Research Center, Lund University, Skåne University Hospital,

Jan Waldenströms gata 35, 205 02 Malmö, Sweden

Figure legends

Supplementary fig. S1

A, Bar graphs showing alteration in transcript level expression of *CD274*, *CYSLTR1*, and *CTNNB1* in HT-29 colon cancer cells stimulated with IFNγ (50 ng/mL) for 24 h.
Mean ± SEM. ***P < 0.001, two-tailed unpaired t-test.

Supplementary fig. S2

XY scatter plots showing the correlation between *CYSLTR1*, and *IFNG* transcript levels in the **A,** TCGA-COAD (n = 328), and **B,** GSE39582 (n = 585) datasets of colon cancer patients.
Immunofluorescence images showing PD-L1 expression following treatment of **C,** SW480 cells with IFNγ (50 ng/mL). Bars, 10 µm.
D, Bar graph showing the mRNA expression of *CYSLTR1* in Dox-inducible conditionally knock-down of *CYSLTR1* in HCT116 cells exposed to either IFNγ or left untreated.
Mean ± SEM. **P < 0.01, ***P < 0.001, two-tailed unpaired t-test.

Supplementary fig. S3

A, Western blots showing alterations in the expression of the indicated proteins in IFNγ-stimulated (50 ng/mL, 24 h) HT-29 CC cells subsequently treated with either Montelukast (Mo) or in a combination with GSK-3β inhibitor, CHIR-99021 (10 µM; 24 h) (Mo + CHIR-99021). The blots are representative of three replicates, which are shown in the densitometry graph.

B, Western blots showing alterations in the expression of the indicated proteins in IFNγ-stimulated (50 ng/mL, 24 h) HT-29 CC cells transfected with *CRISPR/Cas9-CTRL* or

CRISPR/Cas9-CYSLTR1 subsequently treated with GSK-3β inhibitor, CHIR-99021. The blots are representative of three replicates, which are shown in the densitometry graph.

In all the western blot panels, GAPDH served as the loading control. Mean ± SEM. *P < 0.05, **P < 0.01, two-tailed unpaired t test. MW, relative molecular weight expressed in kilodaltons (kDa).

Supplementary fig. S4

A, q-RT-PCR analysis of the relative mRNA expression of *CD274* in HT-29 cell xenografts and SW480 cell xenografts in mice treated with DMSO or montelukast (Mo).

B, Immunofluorescence images showing the expression of β-catenin (green) and PD-L1 (red) in SW480 cell xenografts in mice treated with DMSO or Montelukast (Mo). Representative graph **B’,** showing the MFI of PD-L1 expression (n = 4 per group in each xenograft). Scale bars as indicated in the images. The white dotted line marks the outline of the xenograft section.

Western blots of **C,** SW480 cell xenograft. The blots show the expression of the indicated proteins of interest. Representative densitometric analysis results are shown in the graph in **C’**, for SW480 cells.

In all the western blot panels, GAPDH served as the loading control. The MFI in all confocal images was calculated with ImageJ software (NIH, USA). Mean ± SEM. **P < 0.01, ***P < 0.001, two-tailed unpaired t test. MW, relative molecular weight expressed in kilodaltons (kDa).

Supplementary fig. S5

A, Bar graphs showing alteration in transcript level expression of *CD274*, *CYSLTR1*, and *CTNNB1* in HT-29 colon cancer cells stimulated with IFNγ (50 ng/mL) followed by either treated with Montelukast (Mo, 10 μM), Atezolizumab (PD-L1 neutralizing antibody) alone or in the combination of both (Mo + Atezolizumab).

Mean ± SEM. *P < 0.05, **P < 0.01, two-tailed unpaired t-test.

Supplementary fig. S6

A, Western blots showing alterations in the expression of the indicated proteins in the nuclear or cytoplasmic fraction of IFNγ-stimulated (50 ng/mL, 24 h) SW480 CC cells subsequently treated with either Montelukast (Mo) or Atezolizumab alone or in a combination of Montelukast and Atezolizumab. The blots are representative of three replicates, which are shown in densitometry graphs.

In the western blot panels, GAPDH or LAMIN B1 served as the loading control. Mean ± SEM. *P < 0.05, **P < 0.01, two-tailed unpaired t test. MW, relative molecular weight expressed in kilodaltons (kDa).

Supplementary fig. S7

A, Box plot showing *CD274* transcript level expression in normal (n = 41) and cancer (n = 286) tissue in the TCGA-COAD dataset (n = 327).

Mean ± SEM. *P < 0.05, ***P < 0.001, two-tailed unpaired t-test.

Supplementary fig. S1

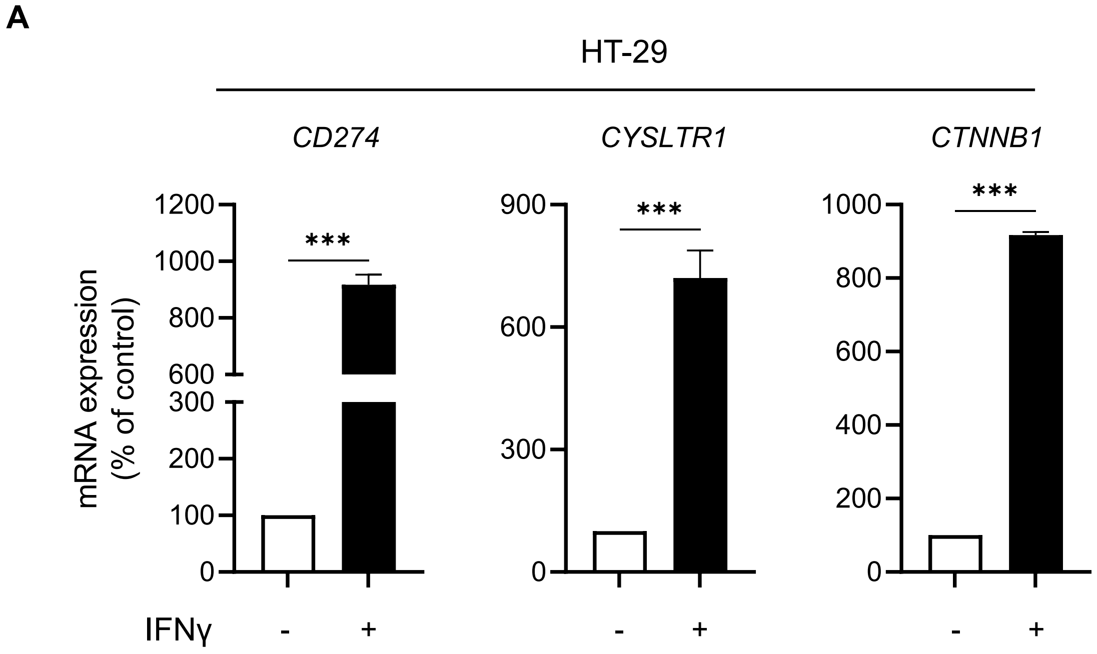

Supplementary fig. S2

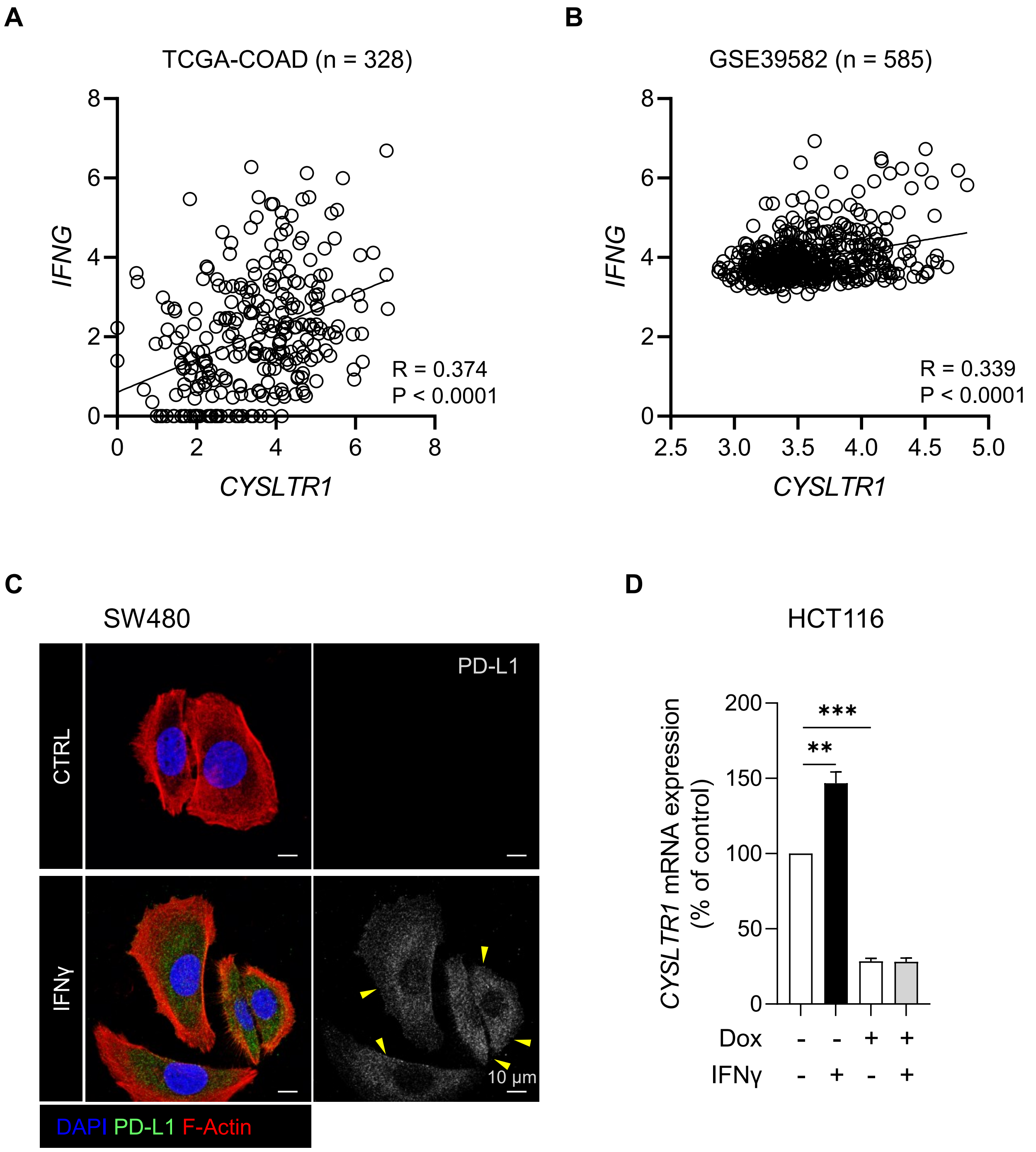

Supplementary fig. S3

A

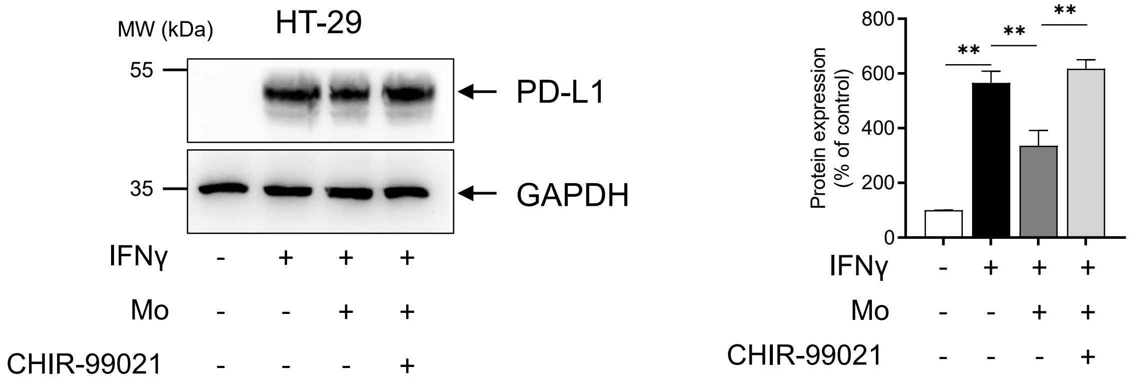

B

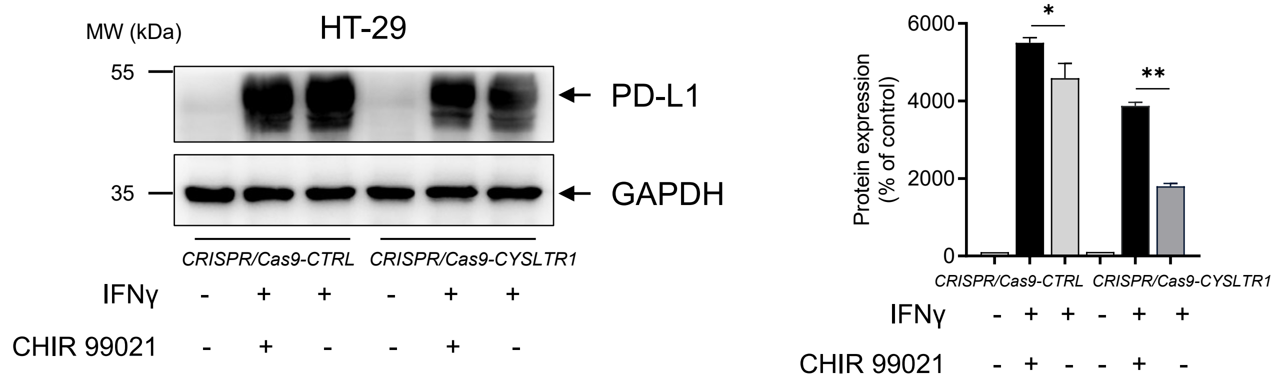

Supplementary fig. S4

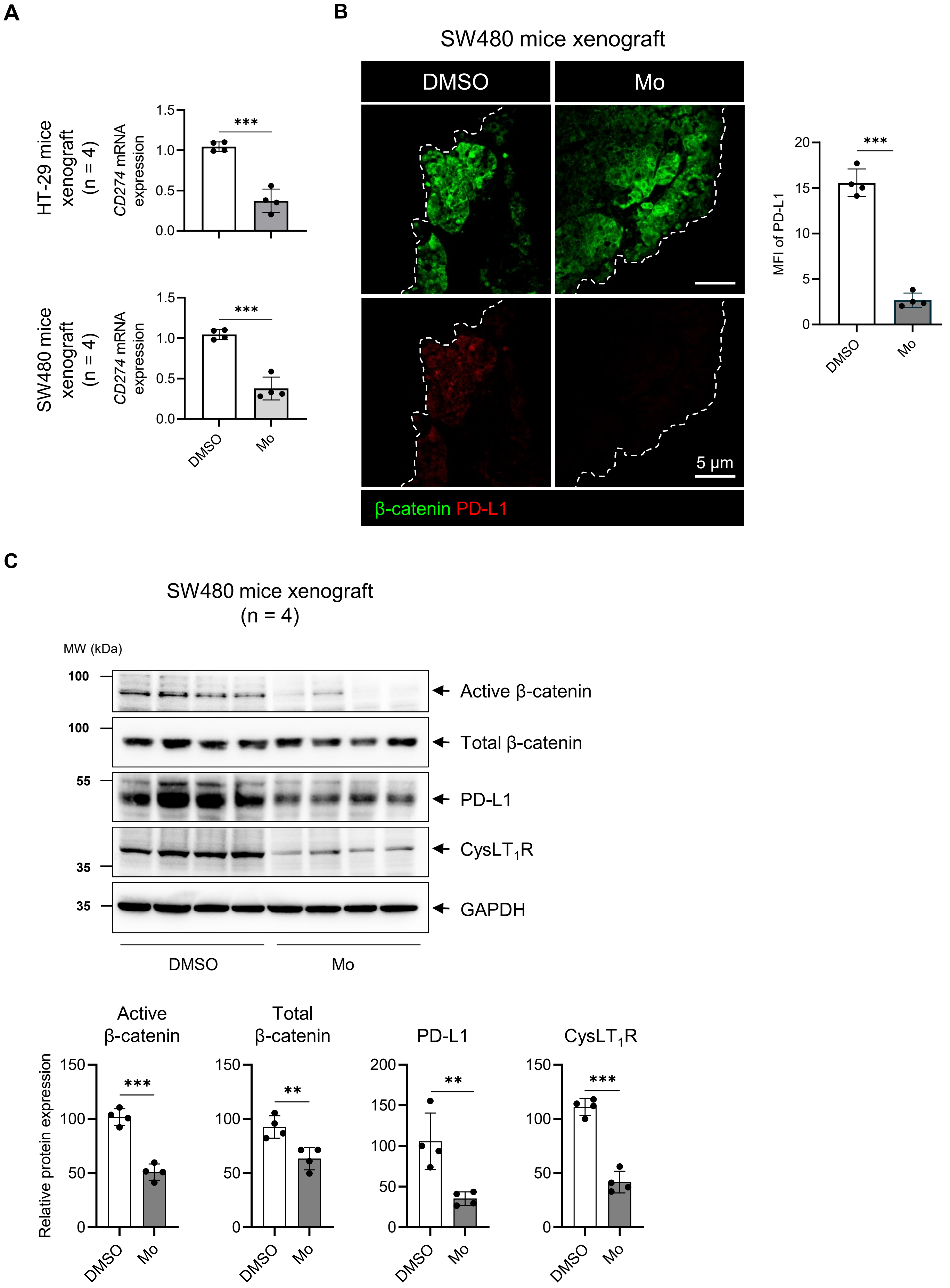

Supplementary fig. S5

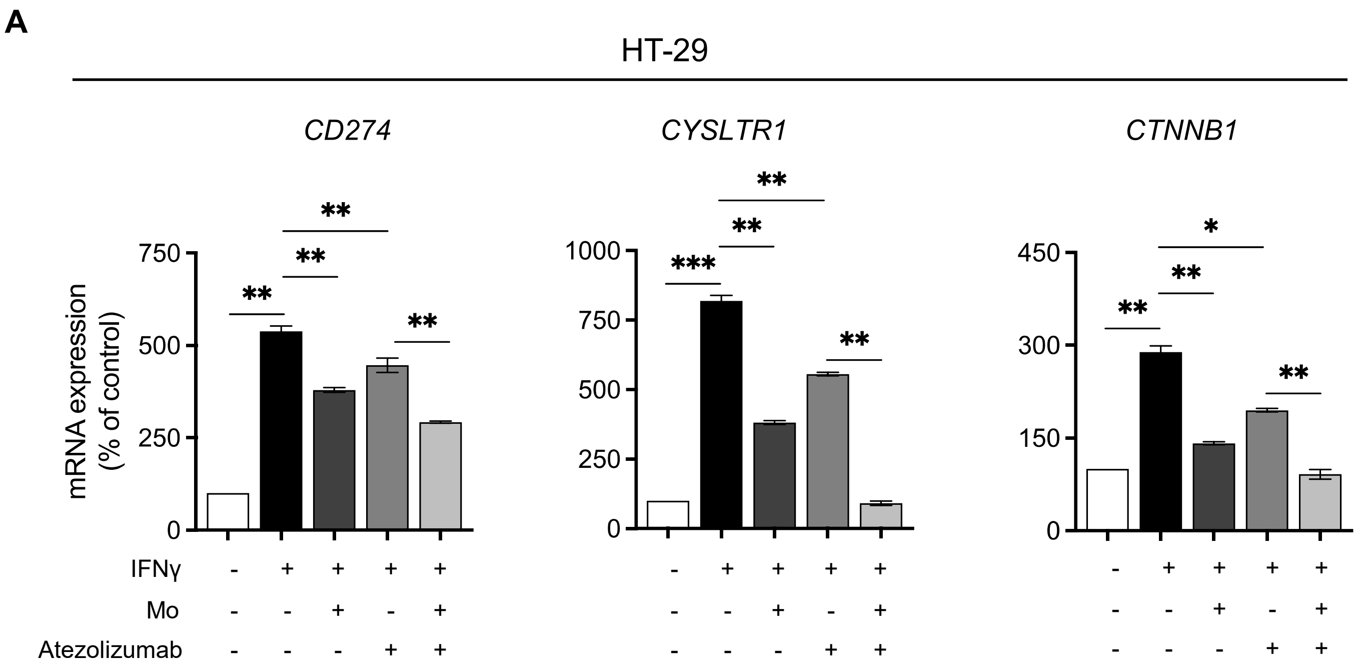

Supplementary fig. S6

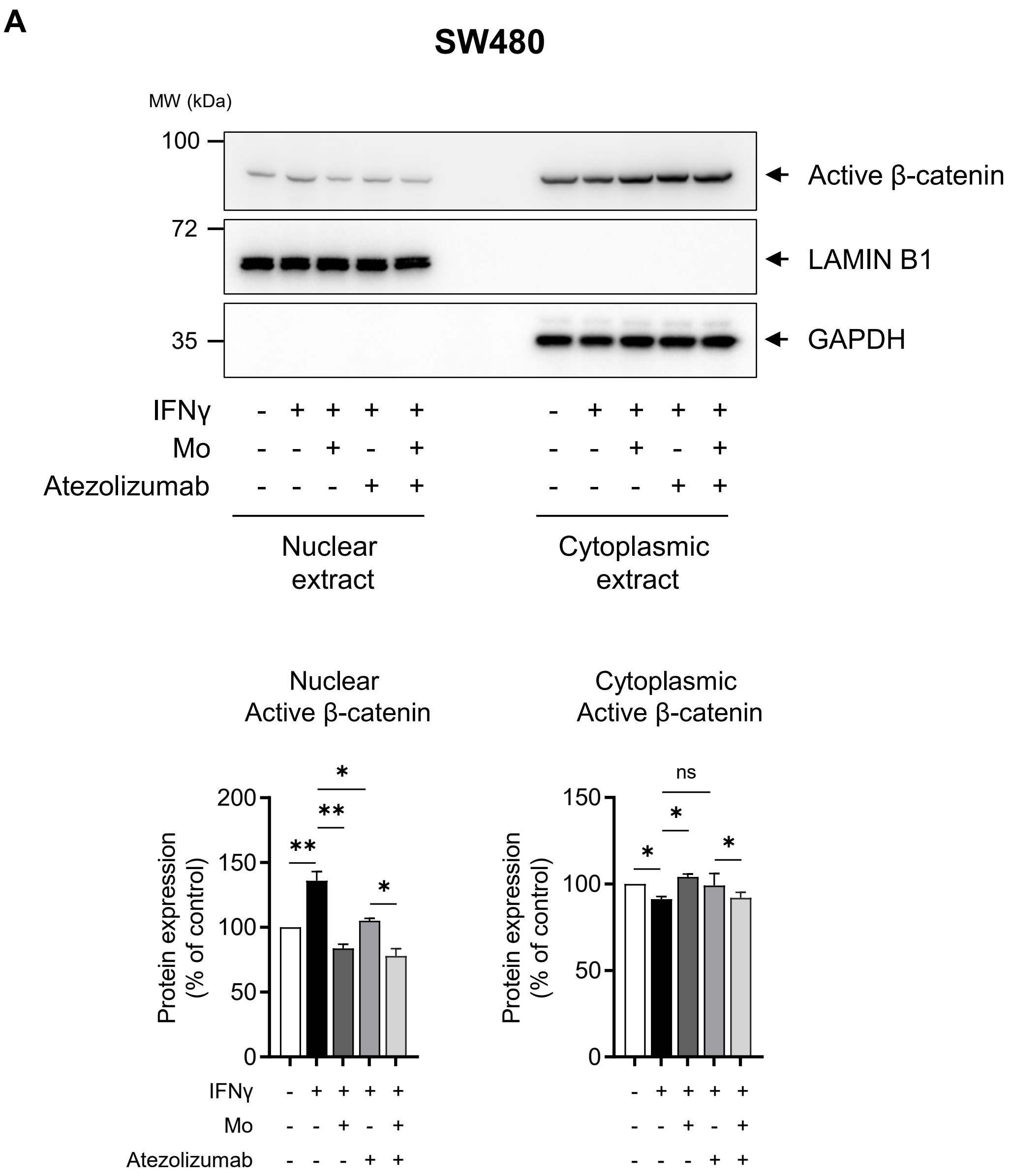

Supplementary fig. S7

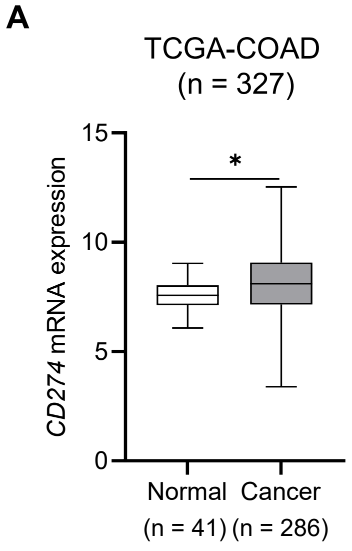

Supplement: Supplementary file 2 — Additional file 1. [file 12964_2023_1157_MOESM1_ESM.pdf]
